# Hyperaesthesia Following Genital Herpes: A Case Report

**DOI:** 10.1155/2011/903595

**Published:** 2011-04-18

**Authors:** Catriona Ooi, Vijay Zawar

**Affiliations:** ^1^Sexual Health Physician, Pacific Clinic, 670 Hunter Street, Newcastle, NSW 2300, Australia; ^2^Skin Diseases Center, Nashik 422001, Maharashtra State, India

## Abstract

We report an adult female patient who presented with sacral radiculopathy as incapacitating dysthesias following primary genital herpes simplex, which later recurred. Despite use of systemic antiviral treatment, the painful syndrome in our patient persisted. The success in treatment was seen only after the addition of amitriptyline hydrochloride. The case is being presented here for its rare manifestation and novel use of amitriptyline hydrochloride.

## 1. Case Presentation

We present the case of a 64-year-old female teacher who presented to the sexual health clinic in Newcastle, Australia. 

Six weeks previously, the patient had attended her primary care doctor (PCD) with dysuria and was treated for a urinary tract infection; however, symptoms persisted. Several days later she noticed acute onset of bilateral vulval blisters. She was clinically diagnosed with genital herpes and commenced on valacyclovir 500 mg bd for one week. The blisters resolved within five days; however, she developed burning and exquisite sensitivity to touch in both inner thighs and in the left buttock extending down the left leg and including the sole of the left foot and toes. She found that at times, she was unable to walk and sit. She returned to her PCD who continued valacyclovir for another week; however, the burning persisted.

On presentation to the sexual health clinic, the patient had completed six weeks of valacyclovir 500 mg bd with minimal improvement. Vulval ulcers had not recurred. The patient denied any other signs or symptoms and gave no history of prodromal symptoms. She was in a monogamous sexual relationship of four months duration with a male partner who was taking suppressive treatment for herpes simplex type 2 (HSV2) genital infection. She was unable to comment regarding her partners' adherence with this treatment. They did not use condoms consistently. Her previous sexual partner, over 12 months previously, was her husband for 20 years. The patient had no prior history of genital herpes, and her PCD was not able to be contacted to confirm swab collection or diagnosis. General history was unremarkable; she had a total hysterectomy at age 40 with sparing of the ovaries and denied history of shingles; however, she had chicken pox as a child. She had commenced escitalopram oxalate 10 mg/d at the time of her marriage breakup four months previously.

Examination of the genital area was unremarkable. Neurological examination of the lower limbs revealed hyperaesthesia in dermatomes S1, S2, and S3 on the left side. There was no other sensory abnormality or motor deficit found. A herpes virus polymerase chain reaction (PCR) swab test at the site of healed vulval blisters was negative for both varicella zoster virus and herpes simplex virus (HSV). First catch urine PCR was negative for *Neisseria gonorrhoea* and *Chlamydia trachomatis*. The patient declined serology for herpes simplex infection. Pain management options were discussed including low-dose tricyclic antidepressants, gabapentin, and simple analgesics. 

The patient returned four days later and was started on amitriptyline hydrochloride 10 mg nocte increased to 20 mg nocte after two weeks. Potential interaction with escitalopram oxalate was discussed, and the patient was advised that this was low. The pain resolved after four weeks of amitriptyline hydrochloride at 20 mg, and the treatment was ceased. 

The patient experienced a further episode of genital blisters three months later and self-medicated with topical acyclovir. Vulval lesions resolved within two days; however, she again experienced residual neuropathic pain in the same distribution. This episode was milder than the first and responded after three weeks of amitriptyline hydrochloride treatment. 

Genital herpes is commonly due to HSV2; however, an increasing proportion of new infections are caused by HSV1, primarily responsible for orolabial infection. In fact, either HSV1 or 2 may infect genital or extra genital sites. Although unconfirmed microbiologically, this patient gives a classic history of first episode genital herpes complicated by sacral radiculopathy manifesting as hyperaesthesia. Vulval PCR swab taken at our clinic was negative. This was collected despite complete healing of lesions, as PCR testing may detect asymptomatic viral shedding although sensitivity is low. As the patient was still experiencing hyeraesthesia, and this was the first episode infection, it was postulated that she may have continued to shed virus. However, given the previous few weeks of antiviral medications, this test was predictably negative. 

At 64 years, this patient is significantly older than most patients presenting to health services with first episode herpes, the median age being mid 20s [1, 2]. Her low-risk sexual history may be one explanation. Given her current partner is HSV2 positive, this is most likely the causative virus. Once infected, the virus establishes latency in the dorsal root ganglion, asymptomatically shedding virus, and reactivating in the form of recurrent lesions from time to time. Studies have shown that HSV2 recurs more frequently than HSV1, and although suppressive antiviral treatment will decrease frequency of recurrent infection and asymptomatic shedding, it does not prevent it. Furthermore, antiviral treatment has shown to decrease rates of partner transmission [3]. Similarly, condoms, even if used consistently are not 100% reliable although they offer good protection [4]. Condoms were not used consistently by our patient.

First-episode infections are associated with more extensive disease, systemic symptoms, and greater viral shedding than recurrences [5]. Patients experiencing first episode genital herpes classically present with pain, itching, and burning, with 80% of women reporting dysuria [6]. Although systemic symptoms are present in 70% of women, they were absent in this case. 

Neurological complications are typically associated with primary infection; however, unlike aseptic meningitis and accompanying meningism which occurs commonly in an estimated 20%–30% of individuals, autonomic nervous system dysfunction and sacral radiculopathy is less common, estimated in 1%-2% of cases [6, 7]. Peripheral neurological complications associated with autonomic nervous system involvement have been described in the literature and vary enormously from paraesthesias, hyperaesthesias, and dysthesias to urinary retention and constipation. Transverse myelitis has also been reported, and in some cases it has been fatal [8]. Myelitis occurring together with autonomic nervous system involvement presents as urinary retention as a result of sacral myeloradiculitis and is known as *Elsberg syndrome* [9]. 

Whilst most case reports are in patients diagnosed with HSV2, peripheral neurological complications may occur with either HSV type [10, 11]. These usually resolve gradually over days to weeks, but as demonstrated in this case, symptoms can persist for months [5, 12]. While case reports have suggested that effective antiviral medication provides moderate relief and speeds recovery, we did not observe a significant effect of pain moderation in our patient [13, 14].

Neuropathic pain commonly occurs with recurrences in the prodromal phase of infection. Reactivation of infection results in neuropathic symptoms at the potential lesions site or along the distribution of the infected ganglia. It is uncommon to experience neurological complications of recurrent infection although these have been described [15]. However, there are few reports describing an association of herpes simplex infection and hyperaesthesia.

Neurological complications are more frequently seen with varicella zoster virus (VZV), another alpha virus of the *herpesviridae* family. Although there are various definitions of post herpetic neuralgia (PHN), it is estimated that 3%–7% of patients have pain persisting for more than three months following shingles rash, 2%–5% after one year [16–19]. The pathogenesis of PHN and similar neuropathic pain syndromes is due to inflammation, neuronal damage, and dysregulation of the immune system; although, the actual mechanism remains unclear. Studies have shown that the incidence and duration of PHN are associated with age [20]. Age may have been a significant factor in our 64-year-old patient. We considered the diagnosis of PHN complicating vulval zoster in the patient. Unlike HSV, however, VZV reactivations are rare in immunocompetent individuals due to the differences in molecular mechanisms controlling latency of these viruses [21].

Patient clinical and medical history, together with the absence of lesions in the orolabial area and elsewhere, helped to exclude other differential diagnoses such as drug eruptions (fixed drug eruptions, erythema multiforme), pemphigus, the pemphigoid group of diseases, insect bites, and infestations. The recurrent nature patient symptoms, typical of genital herpes, helped to confirm this.

In this case, valacyclovir had minimal effect on neurological pain despite prompt resolution of vulval lesions. Antiviral medication has been shown to speed healing of the VZV rash and reduce viral shedding and new lesion formation. As a result of reduced viral replication, antiviral treatment may limit initial neuronal damage and inflammation, thereby ameliorating neuropathic complications. Antiviral influence on clinical course is unclear in this case. Valacyclovir was commenced approximately one week following symptom onset (i.e., at least one week of viral replication). We are unable to speculate as to the influence of this medication. 

As hyperaesthesia prevented the patient from working we elected to treat with low-dose tricyclic antidepressants (TCAs). TCAs, at low doses, have shown to be effective in treatment of neuropathic pain by blocking the reuptake of monoamine neurotransmitters released by descending axons from the brainstem, thereby augmenting catecholamine neurotransmission. Experience is mostly with amitriptyline hydrochloride, which has been studied extensively for use in PHN. Used in combination with antiviral medication, low-dose amitriptyline hydrochloride has been shown to significantly decrease duration of PHN in patients >60 years [22]. While TCAs use in PHN is well documented, there are no documented cases we could find for its use in hyperaesthesia complicating genital herpes. Although the patient reported an improvement in symptoms, it is unknown whether use of amitriptyline hydrochloride affected the initial clinical course; however, prompt response to recurrent hyperaesthesia is more convincing. Certainly, with treatment, she was able to return to work. 

The major weakness in this case is that we were unable to make a definitive laboratory diagnosis. However, the classic and recurrent nature of the lesions, history of definite exposure to a HSV2 positive source, and prompt lesion healing with the use of antiviral medication lead us to strongly believe that the clinical diagnosis was herpes simplex infection in both the episodes. 

In conclusion, sacral radiculopathy may present as troublesome hyperesthesia following primary genital herpes simplex infection. Amitriptyline may offer a therapeutic option in such patients if systemic antiviral therapy is inadequate or ineffective.

## References

[1] Richards J, Krantz E, Selke S, Wald A (2008). Healthcare seeking and sexual behavior among patients with symptomatic newly acquired genital herpes. *Sexually Transmitted Diseases*.

[2] Sexually transmitted diseases quarterly report: genital warts and genital herpes simplex virus infections in England and Wales 1994.

[3] Corey L, Wald A, Patel R (2004). Once-daily valacyclovir to reduce the risk of transmission of genital herpes. *The New England Journal of Medicine*.

[4] Wald A, Langenberg AGM, Link K (2001). Effect of condoms on reducing the transmission of herpes simplex virus type 2 from men to women. *Journal of the American Medical Association*.

[5] Corey L, Wald A, Holmes KK, Sparling FP, Mardh PA (1999). Genital herpes. *Sexually Transmitted Diseases*.

[6] Corey L, Adams HG, Brown ZA, Holmes KK (1983). Genital herpes simplex virus infections: clinical manifestations, course, and complications. *Annals of Internal Medicine*.

[7] Brugha R, Keersmaekers K, Renton A, Meheus A (1997). Genital herpes infection: a review. *International Journal of Epidemiology*.

[8] Wiley CA, VanPatten PD, Carpenter PM, Powell HC, Thal LJ (1987). Acute ascending necrotizing myelopathy caused by herpes simplex virus type 2. *Neurology*.

[9] Berger JR, Houff S (2008). Neurological complications of herpes simplex virus type 2 infection. *Archives of Neurology*.

[10] Shyu WC, Lin JC, Chang BC, Harn HJ, Lee CC, Tsao WL (1993). Recurrent ascending myelitis: an unusual presentation of herpes simplex virus type 1 infection. *Annals of Neurology*.

[11] Haanpää M, Paavonen J (2004). Transient urinary retention and chronic neuropathic pain associated with genital herpes simplex virus infection. *Acta Obstetricia et Gynecologica Scandinavica*.

[12] Sasadeusz JJ, Sacks SL (1994). Herpes latency, meningitis, radiculomyelopathy and disseminated infection. *Genitourinary Medicine*.

[13] Eberhardt O, Küker W, Dichgans J, Weller M (2004). HSV-2 sacral radiculitis (Elsberg syndrome). *Neurology*.

[14] Kallio-Laine K, Seppänen M, Lokki ML (2008). Widespread unilateral pain associated with herpes simplex virus infections. *The Journal of Pain*.

[15] Whitley RJ, Kimberlin DW, Roizman B (1998). Herpes simplex viruses. *Clinical Infectious Diseases*.

[16] Hope-Simpson RE (1965). The nature of herpes zoster: a long-term study and a new hypothesis. *Proceedings of the Royal Society of Medicine*.

[17] Ragozzino MW, Melton LJ, Kurland LT (1982). Population-based study of herpes zoster and its sequelae. *Medicine*.

[18] Helgason S, Sigurdsson J, Gudmundsson S (1996). The clinical course of herpes zoster: a prospective study in primary care. *European Journal of General Practice*.

[19] Choo PW, Galil K, Donahue JG, Walker AM, Spiegelman D, Platt R (1997). Risk factors for postherpetic neuralgia. *Archives of Internal Medicine*.

[20] Decroix J, Partsch H, Gonzalez R (2000). Factors influencing pain outcome in herpes zoster: an observational study with valaciclovir. *Journal of the European Academy of Dermatology and Venereology*.

[21] Cohen JI, Brunell PA, Straus SE, Krause PR (1999). Recent advances in varicella-zoster virus infection. *Annals of Internal Medicine*.

[22] Bowsher D (1997). The effects of pre-emptive treatment of postherpetic neuralgia with amitriptyline: a randomized, double-blind, placebo-controlled trial. *Journal of Pain and Symptom Management*.

